# Development and Validation of a Risk-Score Model for Type 2 Diabetes: A Cohort Study of a Rural Adult Chinese Population

**DOI:** 10.1371/journal.pone.0152054

**Published:** 2016-04-12

**Authors:** Ming Zhang, Hongyan Zhang, Chongjian Wang, Yongcheng Ren, Bingyuan Wang, Lu Zhang, Xiangyu Yang, Yang Zhao, Chengyi Han, Chao Pang, Lei Yin, Yuan Xue, Jingzhi Zhao, Dongsheng Hu

**Affiliations:** 1 Department of Preventive Medicine, Shenzhen University School of Medicine, Shenzhen, Guangdong, People’s Republic of China; 2 Department of Epidemiology and Health Statistics, College of Public Health, Zhengzhou University, Zhengzhou, Henan, People’s Republic of China; 3 Department of Prevention and Health Care, Military Hospital of Henan Province, Zhengzhou, Henan, People’s Republic of China; Florida International University Herbert Wertheim College of Medicine, UNITED STATES

## Abstract

Some global models to predict the risk of diabetes may not be applicable to local populations. We aimed to develop and validate a score to predict type 2 diabetes mellitus (T2DM) in a rural adult Chinese population. Data for a cohort of 12,849 participants were randomly divided into derivation (n = 11,564) and validation (n = 1285) datasets. A questionnaire interview and physical and blood biochemical examinations were performed at baseline (July to August 2007 and July to August 2008) and follow-up (July to August 2013 and July to October 2014). A Cox regression model was used to weigh each variable in the derivation dataset. For each significant variable, a score was calculated by multiplying β by 100 and rounding to the nearest integer. Age, body mass index, triglycerides and fasting plasma glucose (scores 3, 12, 24 and 76, respectively) were predictors of incident T2DM. The model accuracy was assessed by the area under the receiver operating characteristic curve (AUC), with optimal cut-off value 936. With the derivation dataset, sensitivity, specificity and AUC of the model were 66.7%, 74.0% and 0.768 (95% CI 0.760–0.776), respectively. With the validation dataset, the performance of the model was superior to the Chinese (simple), FINDRISC, Oman and IDRS models of T2DM risk but equivalent to the Framingham model, which is widely applicable in a variety of populations. Our model for predicting 6-year risk of T2DM could be used in a rural adult Chinese population.

## Introduction

The prevalence of diabetes, especially Type 2 diabetes mellitus (T2DM), is growing at a worrying rate in the world. In 2013, 382 million people had diabetes worldwide, and this number is expected to increase to 592 million by 2035 [1]. About 80% of people with diabetes are in low- and middle-income countries [1]. As a developing country, China is inevitably faced with a serious prevalence of this disease. In 2013, China had a large burden of diabetes: 1 in 4 people had the disease [2]. This disease may reduce life expectancy by about 10 years [3]. Thus, T2DM is a major public health problem, causing a significant burden on patients, their families, and society.

Although the mechanisms of T2DM remain unclear, people with T2DM are usually asymptomatic in the early period. Several studies have demonstrated that T2DM can be prevented with a vast array of interventions in people at high risk [4–6]. Therefore, prevention among high-risk individuals is an attractive and practical approach to reduce the prevalence of T2DM [7].

A number of diabetes risk-score models have been developed to predict the risk of T2DM [8–11]. These models can be used in clinical practice to identify people at high risk of T2DM and to guide clinical treatment. Some national and international diabetes guidelines have recommended diabetes risk-assessment tools as a simple screening method for identifying people who may be at high risk [12–14]. However, whether these models can be applied to local populations is not ensured. Indeed, the incidence and risk factors of T2DM in a population determine the suitability of a risk score. Some scores developed in a particular population often do not perform well in other populations [15].

Here, we developed and validated a prediction model for T2DM in a cohort of rural adult Chinese people.

## Materials and Methods

### Study design and participants

In total, 20,194 participants ≥18 years old were recruited from a rural Chinese population from July to August of 2007 and July to August of 2008 (baseline); 17,262 (85.5%) were followed up from July to August 2013 and July to October 2014. The same questionnaire interview and physical and blood biochemical examinations were performed at baseline and follow-up. We excluded people lost to follow-up (n = 2932), who had a diagnosis of T2DM at baseline (n = 1230), had unknown T2DM at follow-up (n = 2083) or died during follow-up (n = 1100). Data for 12,849 participants were selected for this analysis and were randomly divided into derivation (n = 11,564) and validation (n = 1285) datasets to establish and validate the model. Randomization was carried out by use of random numbers generated by computer.

The study was approved by the Ethics Committee of Zhengzhou University School of Medicine, and all participants provided informed written consent.

### Data collection

Trained investigators administered a questionnaire (collecting data on demographic characteristics, dietary and lifestyle behaviors, family history of T2DM). Education level was categorized as no education, elementary level, secondary school, high school, and college and above. Marital status was classified as married/cohabitating and unmarried/divorced/widowed. The daily food intake composition was calculated according to the China Food Composition Table [16]. The limits of high-fat and high-vegetable consumption were 30 g/d and 500 g/d, respectively, based on the Dietary Guidelines for Chinese Residents [17]. Smoking was defined as currently smoking and/or having smoked at least 100 cigarettes during the lifetime. Drinking was defined as having consumed at least 30 g of alcohol per week in the previous year. According to the International Physical Activity Questionnaire (IPAQ) [18], physical activity level was classified as low, moderate, or high. Family history of T2DM was considered positive with either parent having a history of T2DM.

Body weight, height and waist circumference (WC) were measured by standard methods [19]. Body mass index (BMI) was calculated by mass in kilograms divided by height in meters squared [20]. An electronic sphygmomanometer (OMRON HEM-7071, Japan) was used to measure blood pressure and heart rate (HR). Pulse pressure (PP) was calculated as systolic blood pressure minus diastolic blood pressure. Overnight fasting blood samples were collected in a vacuum tube with disodium EDTA and centrifuged at 3000 rpm for 10 min, then plasma was transferred to an EP tube and stored at -20°C for blood biochemical examination. Levels of fasting plasma glucose (FPG), total cholesterol (TC), triglycerides (TG) and high-density lipoprotein-cholesterol (HDL-C) were detected by using an automatic biochemical analyzer (Hitachi 7080, Tokyo) with reagents from Wako Pure Chemical Industries (Osaka, Japan). Low-density lipoprotein-cholesterol (LDL-C) level was calculated by the Freidwald formula [21].

### Definition of T2DM

According to the Guideline for prevention and treatment of type 2 diabetes in Chinese (2013 edition) [22], T2DM was diagnosed by FPG ≥7.00 mmol/L and/or current treatment with anti-diabetes medication. We excluded subjects with type 1 diabetes, gestational diabetes and other diabetes types.

### Statistical analyses

We used covariates of T2DM risk ascertained from the literature: gender, age, educational level, marital status, smoking, drinking, high-fat diet, high-vegetable diet, physical activity, family history of T2DM, BMI, WC, PP, HR and levels of TC, TG, HDL-C, LDL-C and FPG. In comparing the derivation and validation datasets at baseline, the Mann-Whitney Wilcoxon test was used for continuous variables because of non-normal distribution and chi-square test for categorical variables. Person-years of follow-up and the incidence density rate were computed.

Disease-free survival was analyzed by the Kaplan-Meier method, with the log-rank test to compare survival curves. A Cox proportional-hazards model with forward selection was used for multivariable survival analysis. Coefficients (β) and baseline hazard function [*h*_*0*_(*t*)] were estimated by Cox regression analysis. For each variable significant on Cox regression analysis, a score was calculated by multiplying β by 100 and rounding to the nearest integer. The total score was the sum of scores for each factor. [*h*_*0*_(*t*)] was T2DM-free average survival probability at time t (e.g., *t* = 6 years). The probability (*P*) of T2DM over 6 years was calculated as follows:

$$P(T2DM)=1-h_{0}{\left(t\right)}^{\text{exp}(score/100)}$$

The predictive power of the risk-score model was evaluated to identify the risk of developing T2DM in the derivation and validation datasets. The aggregated scores were divided into four ranges, and the observed 6-year cumulative incidence of T2DM was compared with predicted risk by chi-square test for trend. The model’s accuracy was assessed by the area under the receiver operating characteristic curve (AUC) based on the sum of scores. The AUC performance of the model was compared with that of several prediction models developed in other populations, including the Chinese (simple) [23],FINDRISC [24], Oman [25], IDRS [26] and Framingham [27] models by the DeLong et al. method [28]. The optimal cut-off AUC was defined as having the maximum combination of sensitivity and specificity. Goodness of fit was assessed by the Hosmer–Lemeshow test [29].

Statistical analysis involved use of SAS 9.1 (SAS Institute, Cary, NC) and MedCalc 9.3.1 (Med-Calc, Inc., Mariakerke, Belgium). All statistical tests were two-sided and *P*<0.05 was considered statistically significant.

## Results

### Characteristics of study participants

From the 12,849 participants, we detected 729 in whom T2DM developed during the 6-year follow-up. Overall, the incidence density rate of T2DM was estimated at 9.79/1000 person-years: 9.57 (n = 659) and 9.15/1000 person-years (n = 70) for the derivation and validation datasets, respectively, with no difference between the datasets (*P* = 0.922). The baseline characteristics of subjects did not differ between the two datasets (Table 1).

**Table 1:** Baseline characteristics of subjects in the derivation and validation datasets for developing a model of type 2 diabetes mellitus (T2DM).

Characteristics of subjects	Derivation dataset (*n* = 11,564 subjects)	Validation dataset (*n* = 1285 subjects)	*P* value
Gender (female), n (%)*	7190 (62.18)	819 (63.74)	0.274
**Age (years), median (IQR)** ^**#**^	51 (42, 59)	50 (41, 59)	0.469
Education, n (%)*			0.426
**No education**	1715 (14.83)	171 (13.31)	
**Primary school**	3820 (33.03)	452 (35.18)	
**Middle school**	4868 (42.10)	540 (42.02)	
**High school**	1047 (9.05)	109 (8.48)	
**College and above**	114 (0.99)	13 (1.01)	
Marital status, n (%)*			0.882
**Married/cohabitating**	10628 (91.94)	1182 (92.06)	
**Unmarried/divorced/widowed**	932 (8.06)	102 (7.94)	
High-fat diet, n (%)*	1487 (12.86)	155 (12.06)	0.417
High-vegetable diet, n (%)*	4663 (40.32)	541 (42.10)	0.218
Smoking, n (%)*	2395 (20.71)	266 (20.70)	0.547
Drinking, n (%)*	1294 (11.19)	134 (10.43)	0.410
Physical activity, n (%)*			0.419
**Low**	3253 (28.13)	371 (28.87)	
**Moderate**	2585 (22.35)	302 (23.50)	
**High**	5726 (49.52)	612 (47.63)	
Family history of T2DM, n (%)*	607 (5.25)	73 (5.68)	0.531
**BMI (kg/m**^**2**^**), median (IQR)**^**#**^	24.09 (21.76, 26.59)	24.14 (21.78, 26.64)	0.887
**WC (cm), median (IQR)** ^**#**^	81.75 (74.90, 89.25)	82.05 (75.10, 89.25)	0.708
**PP (mmHg), median (IQR)** ^**#**^	45 (38, 53)	45 (38, 53)	0.750
**HR (bpm), median (IQR)** ^**#**^	74 (67, 81)	73 (67, 80)	0.180
**TC (mmol/L), median (IQR)**^**#**^	4.39 (3.83, 5.01)	4.35 (3.81, 5.02)	0.644
**TG (mmol/L), median (IQR)**^**#**^	1.35 (0.96, 1.95)	1.34 (0.95, 1.93)	0.591
**HDL-C (mmol/L), median (IQR)**^**#**^	1.14 (0.99, 1.32)	1.14 (0.99, 1.32)	0.898
**LDL-C (mmol/L), median (IQR)**^**#**^	2.50 (2.08, 3.00)	2.50 (2.08, 3.00)	0.715
**FPG (mmol/L), median (IQR)**^**#**^	5.32 (4.99, 5.68)	5.31 (4.98, 5.71)	0.540

Data are no. (%) for classification variables and median (IQR) for numeric variables because of a non-normal distribution.

IQR, interquartile range; BMI, body mass index; WC, waist circumference; PP, pulse pressure; HR, heart rate; TC, total cholesterol; TG, triglycerides; HDL-C, high-density lipoprotein cholesterol; LDL-C, low-density lipoprotein cholesterol; FPG, fasting plasma glucose.

*chi-square test.

^#^Mann-Whitney Wilcoxon test.

### Prediction model

Only age, BMI, TG and FPG reached statistical significance and were retained in the Cox regression model with the derivation dataset (Table 2).

**Table 2:** Risk factors of T2DM in the derivation dataset.

Risk factor	β	HR (95%CI)	*P* value	Score allocated
**Age (years)**	0.027	1.027 (1.020–1.034)	<0.001	3
**BMI (kg/m**^**2**^**)**	0.124	1.132 (1.109–1.156)	<0.001	12
**TG (mmol/L)**	0.239	1.270 (1.156–1.396)	<0.001	24
**FPG (mmol/L)**	0.760	1.379 (1.172–1.622)	<0.001	76

HR, hazard ratio; 95% CI, 95% confidence interval; BMI, body mass index; TG, triglycerides; FPG, fasting plasma glucose.

Significant variables were assigned a score based on the regression coefficient (Table 2). The total risk score was calculated as follows:

$$\text{Risk score}=3\times \text{age}(\text{years})+12\times \text{BMI}(\text{kg}/{\text{m}}^{2})+24\times \text{TG}(\text{mmol}/\text{L})+76\times \text{FPG}(\text{mmol}/\text{L})$$

(rounding to the nearest integer for each variable’s score)

The probability (*P*) of T2DM during the 6-year follow-up was calculated by the baseline hazard function [*h*_*0*_(*t*)]:

$$P(T2DM)=1-0.999997^{\text{exp}(score/100)}$$

The probability of T2DM developing in subjects in the derivation dataset was 0.02% to 100% (score 402–1529).

### Evaluation of the model’s predictive performance

The optimal cut-off value for this risk-score model was 936. Sensitivity, specificity and AUC were 66.7%, 74.0% and 0.768 (95% CI 0.760–0.776) with the derivation dataset. To validate the model, we applied this scoring method to the validation dataset. The aggregated scores were divided into 4 ranges (Table 3). For scores of ≤800, 800–899, 900–1099, and ≥1100, the cumulative incidence of T2DM was 1.24%, 2.20%, 9.62%, and 33.13%, respectively, in the derivation dataset and 1.52%, 2.53%, 8.29% and 44.44%, respectively, in the validation dataset. The observed incidence increased with increasing risk score or estimated probability in the 2 datasets (both *P*_trend_ <0.001).

**Table 3:** Estimated probability and observed incidence of T2DM in the derivation and validation datasets.

Score range	Probability, %	Derivation dataset			Validation dataset		
		Non-T2DM, n	T2DM, n	Incidence, %*	Non-T2DM, n	T2DM, n	Incidence, %*
**<800**	<0.89	1830	23	1.24	195	3	1.52
**800–899**	0.89–2.40	4454	100	2.20	501	14	2.53
**900–1099**	2.40–16.44	4435	472	9.62	498	45	8.29
**≥1100**	≥16.44	109	54	33.13	10	8	44.44

T2DM, type diabetes mellitus

*P for trend <0.001.

**Fig 1:**
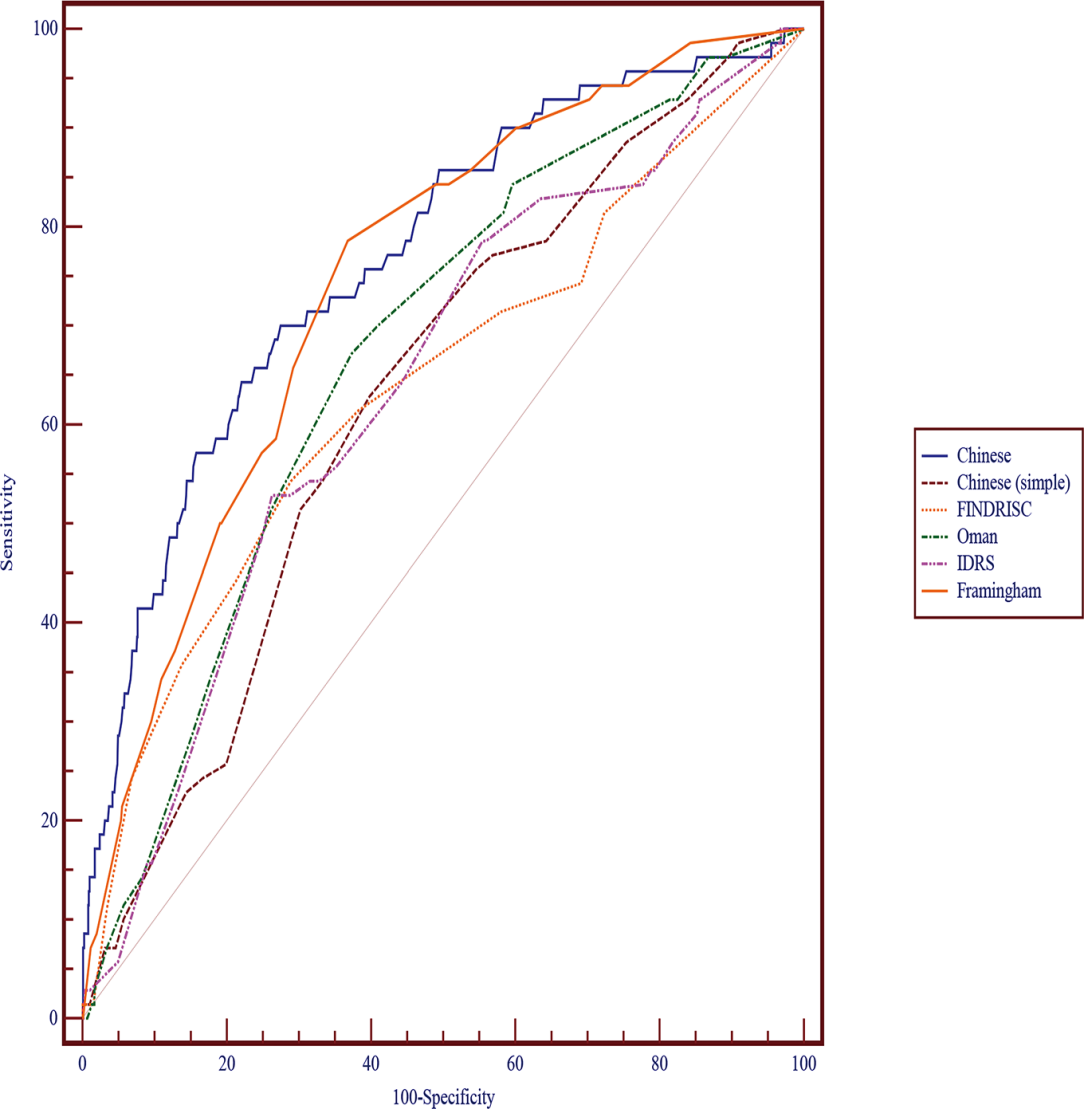
Receiver-operating characteristic (ROC) curves for the Chinese, Chinese (simple), FINDRISC, Oman, IDRS and Framingham models with the validation dataset. Area under the ROC curve: Chinese, 0.766; Chinese (simple), 0.630; FINDRISC, 0.638; Oman, 0.673; IDRS, 0.638; Framingham, 0.745.

Table 4 compares the performance of our model and the Chinese (simple), FINDRISC, Oman, IDRS, and Framingham models with the validation dataset. The AUC was higher for our model than the Chinese (simple), FINDRISC, Oman and IDRS models– 0.766 (95% CI: 0.742–0.789) vs 0.630 (95% CI: 0.603–0.657), 0.638 (95% CI: 0.611–0.664), 0.673 (95% CI: 0.646–0.698) and 0.638 (95% CI: 0.611–0.664)–but not the Framingham model– 0.745 (95% CI: 0.720–0.769). Hence, the performance of our model was superior to the Chinese (simple), FINDRISC, Oman, and IDRS models but equivalent to the Framingham model in a rural adult Chinese population. Moreover, both our model and the Framingham model showed high sensitivity (70.0% and 78.6%), but the specificity was lower for the Framingham than our model (63.2% and 72.5%). The Chinese (simple) model had the lowest AUC of 0.630, and specificity of 60.3%. The FINDRISC and IDRS models had similar performance with the validation cohort, with low sensitivity (54.3% and 52.9%) and high specificity (71.1% and 73.7%). The sensitivity and specificity for the Oman model was 67.1% and 62.7%, respectively. Hosmer-Lemeshow *P* values were non-significant for all models, for satisfactory goodness of fit. Our model had good predicting ability for T2DM (Fig 1).

**Table 4:** Performance of the risk-score model for a rural adult Chinese population (Chinese model) and the Chinese (simple), FINDRISC, Oman, IDRS and Framingham models with the validation dataset.

Model	Optimal cut-off score	Sensitivity (%)	Specificity (%)	AUC (95%CI)	*P* value*	Hosmer-Lesmeshow *P* value^#^
**Chinese**	>936	70.0	72.5	0.766 (0.742–0.789)	-	0.476
**Chinese (simple)**	>13	62.9	60.3	0.630 (0.603–0.657)	<0.001	0.084
**FINDRISC**	>4	54.3	71.1	0.638 (0.611–0.664)	<0.001	0.446
**Oman**	>10	67.1	62.7	0.673 (0.646–0.698)	<0.001	0.345
**IDRS**	>28	52.9	73.7	0.638 (0.611–0.664)	<0.001	0.066
**Framingham**	>10	78.6	63.2	0.745 (0.720–0.769)	0.414	0.177

AUC, area under the receiver operating characteristic curve; 95% CI, 95% confidence interval.

*comparison with the Chinese model.

^#^*P*>0.05 represented better performance.

## Discussion

We aimed to develop and validate a risk-score model for predicting risk of developing T2DM in a rural adult Chinese population. With the model, age, BMI, TG and FPG were predictors of incident T2DM. With the derivation dataset, sensitivity, specificity and AUC were 66.7%, 74.0% and 0.768 (95% CI 0.760–0.776), respectively. With the validation dataset, the performance of our model was superior to the FINDRISC, Oman and IDRS models of T2DM risk but equivalent to the Framingham model, widely used in a variety of populations. Thus, our model for predicting 6-year risk of T2DM could be used for a rural adult Chinese population.

The growth in diabetes incidence is mainly due to the increase in T2DM prevalence [30]. Even so, many cases are still undiagnosed and thus poorly controlled because T2DM has a prolonged latent phase [31–32]. Lifestyle and pharmacological interventions can delay or prevent T2DM in high-risk populations [33–37]. Several randomized clinical trails have demonstrated that interventions can reduce the rate of onset of T2DM in people at high risk of the disease [38–41]. Three follow-up studies showed the rate of conversion to T2DM decreased with lifestyle intervention: 43% reduction over 7 years in the Finnish Diabetes Prevention Study [36], 34% reduction over 10 years in the US Diabetes Prevention Program Outcomes Study [35], and 43% reduction over 20 years in the China Da Qing Diabetes Prevention Study [34]. These findings suggest a promising window in which effective prediction and intervention can lower the prevalence and disease burden of T2DM. Thus, improved efforts are needed to detect people at high risk of T2DM and implement intervention strategies.

Currently, risk prediction models of T2DM are divided into non-invasive and invasive models. Non-invasive risk models are generally based on data obtained by questionnaire and anthropometric measurements for straightforward measurement of T2DM risk. Invasive prediction models are developed on the basis of routine information and laboratory measurements. To obtain sufficient predictive ability, researchers need to include more variables with predictive potential, in some cases even genetic risk factors [42]. A study evaluating the effects of diabetes definitions on diabetes prevalence from a pooled analysis of 96 population-based studies with 331,288 participants, reported that using FPG in population surveys was a strategy for consistent and comparable surveillance [43]. Therefore, we developed an invasive risk-assessment model including FPG.

We established a risk-score model including 4 variables–age, BMI, TG and FPG–based on a rural adult Chinese cohort, to estimate the 6-year probability of developing T2DM (Table 2). The data for these 4 predictors are easy to obtain. The American Diabetes Association (ADA) considers that age is a major risk factor for T2DM and thus recommends the testing of people without other risk factors no later than 45 years old [44]. Age can be used to identify more cases of undiagnosed diabetes when used with the other risk factors in model [45]. However, some researchers suggest that the effect of age on incident T2DM may be mediated by anthropometric measures such as blood pressure, BMI and FPG, which could explain why this factor is not retained in some risk models including these factors [46]. Although age retained in a risk-score model is controversial [11, 27, 47–49], it was a significant factor in our prediction model. However, the association of age and T2DM risk was not overly strong, with an HR of 1.027.

Although our risk-score model showed age as a risk factor of T2DM, this finding does not give much guidance for prevention because age is non-modifiable. The other 3 factors included are meaningful for prevention strategies to reduce the incidence of T2DM. Previous studies found that the modifiable risk factor playing a substantial role on T2DM is obesity [50]. The Nurses’ Health Study, which documented 3300 new cases of T2DM, indicated that BMI, measuring obesity, was a major risk factor for T2DM [51]. TG and FPG are components of metabolic syndrome. Kahn et al. reported that adding information about fasting blood tests could preferably identify people at extreme risk of T2DM with sensitivity 74% and specificity 71% [52]. The Atherosclerosis Risk in Communities study showed that adding data on lipid and fasting blood levels for clinical information can increase AUC values from 0.71 to 0.80 in a model [53]. The Framingham Offspring Study found odds ratios of 1.00 and 1.15 for TG and FPG in predicting 7-year incident T2DM [27]. Our findings are in line with previous results, with hazard ratios of 1.270 and 1.379 for TG and FPG, respectively, in our risk score model.

Because a variable should not be a predictor related to outcome assessment in principle, the inclusion of FPG in the model seemed to not be the case. However, the higher level of FPG might sustained for a substantial time of period, for so-called pre-diabetes. Pre-diabetes is associated with high risk of diabetes developing, with a yearly conversion rate of 5% to 10% [54]. It is an intermediate state of hyperglycemia covering impaired glucose tolerance, impaired fasting glucose or glycated hemoglobin level 6.0% to 6.4% [55]. The ADA indicates that pre-diabetes should not be considered a clinical entity but rather a risk factor of diabetes [44]. Therefore, including FPG as an independent predictor in our model was somewhat reasonable.

Lifestyle changes could prevent T2DM. However, we did not find lifestyle factors such as physical activity, smoking and drinking significant predictors of T2DM in our model after adjusting for other factors, perhaps because of their correlation with BMI, TG and FPG or because data for these factors were not sufficiently accurate as compared with that for the included factors. Similarly, lifestyle factors also contributed less to the model than other variables in the FINDRISC model where the odds ratio of daily consumption of fruits and vegetables and physical activity< 4 h/week were 1.18 (95% CI 0.85–1.64) and 1.31 (95% CI 0.88–1.95) [24].

Validation of a risk-score model often involves comparing estimated probability and observed incidence [8, 56]. We found an overlap between estimated probability and observed incidence, with increased incidence occurring with increasing estimated risk. Thus, estimated risk has a certain accuracy. Comparison with previous prediction models of T2DM using the same dataset can verify the performance of a risk score model. We chose the Chinese (simple) [23], FINDRISC [24], Oman [25], IDRS [26], and Framingham [27] models, with data available from our dataset. Of all the variables, WC, age and family history of diabetes were used to construct the Chinese (simple) score model. Age, BMI, WC, use of blood pressure medication and history of high blood glucose were included for the FINDRISC model from a random population sample of 35- to 64-year-old participants. The Oman model, using Oman’s 1991 National Diabetes Survey data (n = 4881), involved age, WC, family history of diabetes, BMI, and presence of hypertension. The IDRS model, based on a cohort of 10,003 people ≥20 years old in India, involved age, positive family history of diabetes, BMI, WC, and physical activity. The Framingham score system, retaining fasting glucose level, BMI, HDL-C level, parental history of diabetes mellitus, TG, blood pressure or receiving treatment is seminal and widely applicable in a variety of populations [57]. The AUC predictive value of the Framingham model was close to that of our model (0.745 vs 0.766). In general, the Framingham model was more complicated than our model by including seven items. Therefore our model may be more suitable for application in a Chinese population.

Of note, the laboratory variables were not included in the Chinese (simple), FINDRISC, Oman, or IDRS models; therefore, our model having better prediction ability than these models might not be surprising. Similar results were observed in another study [58], finding that the model included both invasive and non-invasive predictors (age, BMI, white blood cell count, TG, HDL-C and FPG) which yielded a higher AUC (0.749) than non-invasive models deriving in America, Europe, or Asia (AUC 0.665–0.703).

Our risk-score model was based on Cox regression for a Chinese rural population. For survival models, the limiting sample size is the number of events if the number of events is smaller than that of nonevents [59]. Peduzzi et al. suggested that a survival model is reasonably stable if the limiting sample size meets a ratio of at least 10 events per variable [60]. The number of T2DM cases was 659 in our derivation dataset. Thus, our sample size is sufficiently large for this type of analysis and limits the problem of over-fitting. Collapsing continuous data into categories results in lost information and power to detect a real relationship, further obtaining optimistic results [59]. Therefore, we retained factors as continuous variables. In addition, our model was developed for a population including young people (≥18 years). T2DM is increasingly common in young people [61], making this an advantage of our model.

Our model has some limitations. First, this is an invasive rather than a non-invasive model, the difficulty and costly for having invasive measurements might restrict its application practically in the rural areas. Second, the cohort study measured only fasting glucose on a single occasion, used to define T2DM at baseline and follow-up. More than three-quarters of the population with impaired glucose tolerance (IGT) and one-third with diabetes with a diagnosis by the 2-h glucose criteria would be classified as normal if they were diagnosed only by fasting glucose [62]. Thus, misclassification bias would be introduced, which would affect estimating the risk of T2DM and hence the performance of model. Future study should aim to develop and validate this risk-score model with 2-h oral glucose tolerance test for diagnosing diabetes. Third, the derivation and validation datasets were from the same cohort. Therefore, the model should undergo external validation for external application. Fourth, a large proportion of participants were lost to follow-up or their diabetic status could not be identified at follow-up. Therefore, the potential bias of lost to follow-up could have been introduced. Finally, it should be noted that the total risk score was larger in our model because a score was calculated by multiplying β by 100 for making better use of the information on age.

## Conclusions

We developed a risk score model to predict T2DM based on age, BMI, TG and FPG for rural adult Chinese people ≥ 18 years old. This model shows adequate performance and may be useful in China to promote the identification of people at high risk of T2DM.
